# Prioritizing the Scale-Up of Evidence-Based Nutrition and Health Interventions to Accelerate Stunting Reduction in Ethiopia

**DOI:** 10.3390/nu11123065

**Published:** 2019-12-16

**Authors:** Kaleab Baye

**Affiliations:** Center for Food Science and Nutrition, College of Natural and Computational Sciences, Addis Ababa University, P.O. Box 1176 Addis Ababa, Ethiopia; kaleabbaye@gmail.com

**Keywords:** stunting, health systems, maternal and child nutrition, growth faltering

## Abstract

Despite some progress, stunting prevalence in many African countries including Ethiopia remains unacceptably high. This study aimed to identify key interventions that, if implemented at scale through the health sector in Ethiopia, can avert the highest number of stunting cases. Using the Lives Saved Tool (LiST), the number of stunting cases that would have been averted, if proven interventions were scaled-up to the highest wealth quintile or to an aspirational 90% coverage was considered. Stunting prevalence was highest among rural residents and households in the poorest wealth quintile. Coverage of breastfeeding promotion and vitamin A supplementation were relatively high (>50%), whereas interventions targeting women were limited in number and had particularly low coverage. Universal coverage (90%) of optimal complementary feeding, preventive zinc supplementation, and water connection in homes could have each averted 380,000–500,000 cases of stunting. Increasing coverage of water connection to homes to the level of the wealthiest quintile could have averted an estimated 168,000 cases of stunting. Increasing coverage of optimal complementary feeding, preventive zinc supplementation, and Water, Sanitation and Hygiene (WASH) services is critical. Innovations in program delivery and health systems governance are required to effectively reach women, remote areas, rural communities, and the poorest proportion of the population to accelerate stunting reduction.

## 1. Introduction

Globally, 155 million children younger than five years of age are stunted [1]. Stunting has been linked to a higher risk of mortality, poor cognitive performance and low productivity and earnings in later life [2,3]. Furthermore, stunting in early life has also been related to a higher risk of chronic diseases in later life [4]. The recognition that most of the growth faltering occurs in the first two years and that the consequences of stunting are largely irreversible past the age of two has led to the first 1000 days movement that spans from the child’s conception to their second birthday [5]. This has led to the development and implementation of various programs that aim to reduce stunting.

Despite remarkable progress, stunting prevalence in many Sub-Saharan African countries remains unacceptably high. For example, Ethiopia has witnessed a rapid stunting reduction from 2000 (60%) to 2016 (38%), but current levels are still very high [6,7]. A much more rapid reduction in stunting will be needed in the upcoming years, if the government’s plan (Sequota Declaration) of achieving zero stunting among children younger than two years of age by 2030 is to be realized. This requires identification of key and effective interventions that, if scaled-up, will result in substantial stunting reduction.

The Lives Saved Tool (LiST) is one such tool that was initially developed to estimate the impact of interventions on mortality of children younger than five years of age [8]. More recently, the tool has been expanded to include nutrition modeling including stunting [9]. Using nationally representative data like the Demographic and Health Surveys (DHS), LiST calculates projected outcomes based on intervention coverage (reach), the fraction of the population affected (e.g., stunted), and the efficacy of the interventions. Such a modeling exercise can help governments prioritize interventions in light of the limited-resources they are consistently confronted with [10,11] and it has also been instrumental in identifying priority interventions highlighted in the Lancet nutrition series [12].

Therefore, the aim of this study was to identify key interventions that can avert the highest number of stunting cases, if scaled-up to the highest wealth quintile or to an aspirational 90% coverage.

## 2. Methods

### 2.1. Modeling Approach Using the LiST

**Figure 1:**
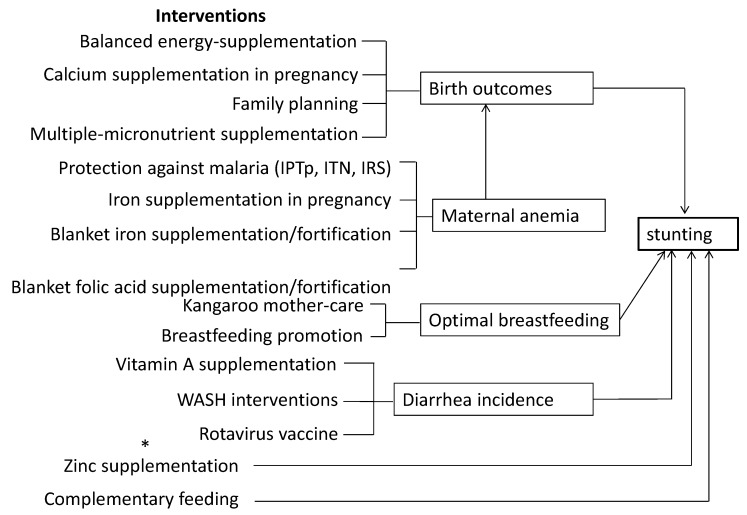
Conceptual framework of key interventions and their link with stunting. * WASH stands for Water, Sanitation and Hygiene.

The LiST model was generated for Ethiopia, taking 2011 as a base year using LiST v5.761. The LiST conceptual framework has been adapted for stunting (Figure 1) [9], and the interventions considered were largely informed by priority interventions highlighted in the Lancet nutrition series [12].

The main outcome of interest was the number of cases of stunting averted. The coverage of key interventions was mainly sourced from nationally representative household surveys such as the Demographic and Health Surveys, which are preloaded in the LiST default database. Updates were made to these pre-loaded coverage data, whenever needed. Based on the most recent available scientific evidence, primarily coming from clinical trials and systematic reviews, the LiST linked coverage to changes in stunting outcomes as follows:

(1) Coverage change x effectiveness x affected proportion = Impact….

As interventions are scaled up, they are used to re-compute projected outcomes on the basis of change in coverage, an estimate of the efficacy of the intervention on stunting, and the affected fraction, which is the proportion of stunting that is likely to be amenable to treatment with the given intervention. More details about the basic calculations and assumption of the LiST and its adaptation to nutritional outcomes can be found in previous publications [8,9]. Details on the data sources for intervention coverage and effectiveness estimates are presented in the Supplementary File S1.

Two scenarios were considered in the modeling approach:

1. An aspirational coverage of 90% that corresponds to a near universal coverage.
2. Coverage to current levels achieved in the wealthiest quintile.

### 2.2. Trends in Stunting Prevalence

The trend in the prevalence of stunting is presented based on nationally-representative estimates using the 2011 and 2016 Ethiopian DHS estimates by wealth quintile and rural/urban residency [6,13]. Equiplots (http://www.equidade.org/equiplot.php) were used to present wealth-quintile and rural/urban inequalities.

### 2.3. Comparison of LiST-Predicted to Observed Changes

Using 2011 as the base year, we compared LiST productions for 2016 to observed prevalence in 2016 DHS. The proportion of change explained by the LiST model was then computed. The LiST equity and missed opportunity tools were used to explore the effect of individually scaling up interventions from current national average to levels of the top wealth quintile and 90%, respectively [14,15]. This helps to determine which intervention scale-up would allow for the greatest number of stunting being averted on the subsequent year. Only interventions that averted more than 5000 cases of stunting are presented.

## 3. Results

**Figure 2:**
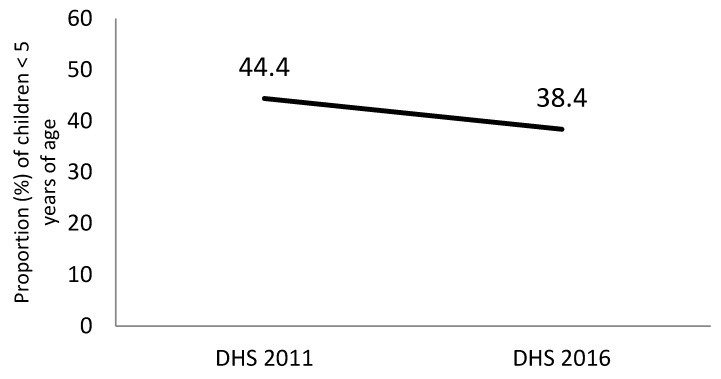
National stunting prevalence among children < 5 years of age in Ethiopia; 2011–2016. DHS, Demographic and Health Survey.

**Figure 3:**
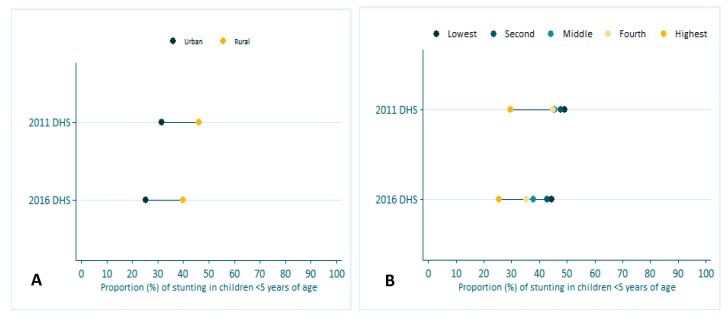
Proportion (%) of stunting in children < 5 years of age in Ethiopia by rural-urban (**A**) and wealth quintile (**B**).

Stunting has gone down by about 6% between 2011 and 2016 (Figure 2). The reductions were observed in both urban and rural areas of Ethiopia (Figure 3A); however, the gap between rural and urban sites has not narrowed, and remained at 15%. In 2011, the difference in stunting prevalence between the highest wealth quintile and the lower wealth quintiles were significantly higher than in 2016 (Figure 3B). However, more than a 20% difference in stunting prevalence is observed between the poorest and the wealthiest quintile.

**Figure 4:**
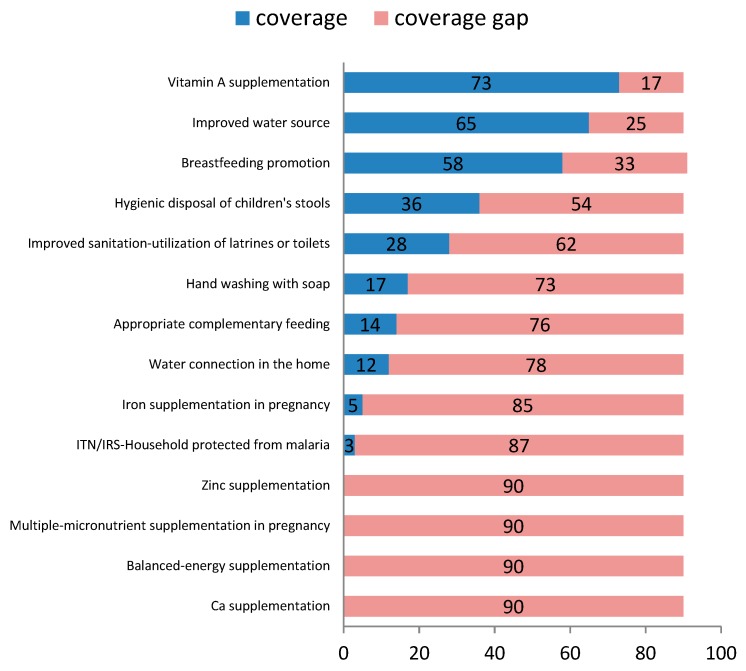
Coverage of effective interventions linked to stunting, 2016.

Among key interventions that are directly or indirectly linked to stunting, the highest coverage was observed for vitamin A supplementation, access to improved water sources, and breastfeeding promotion (Figure 4). Coverage of hygiene- and sanitation-related interventions such as disposal of children’s stool, use of latrines and toilets, and hand washing with soap, all had coverage between 17 and 36%; whereas, water coverage in homes had even lower reach (12%). Only 13.8% of the population had met the minimum dietary diversity score (≥4 food groups), an indicator that served as a proxy for appropriate complementary feeding. Interventions targeting maternal nutrition had low coverage, ranging between 0–5 percent.

**Figure 5:**
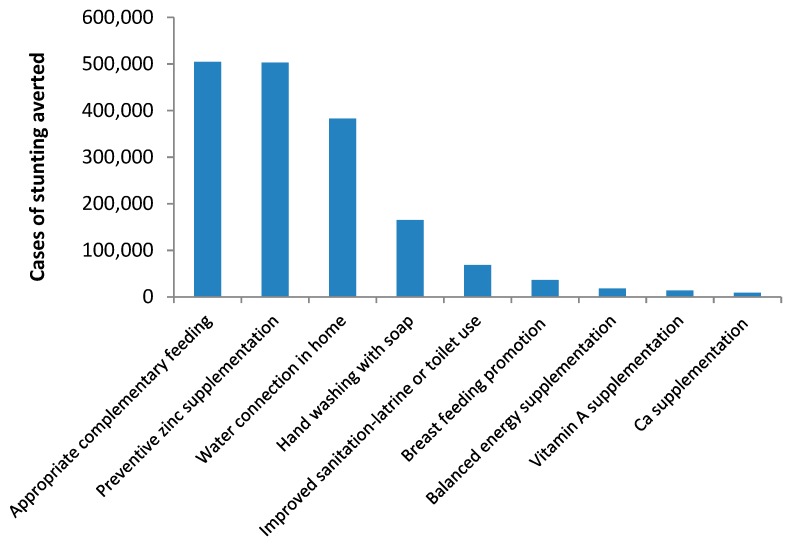
Number of stunting cases that would have been averted with scale-up of key interventions to 90%, 2019.

The biggest missed opportunity comes from the low coverage of appropriate complementary feeding and the absence of preventive zinc supplementation to children (Figure 5). Each of these interventions, if scaled up to 90%, would have averted >500,000 cases of stunting. The model also indicated that increasing coverage of WASH-related interventions would have also averted 67,000 to 383,000 cases of stunting.

**Figure 6:**
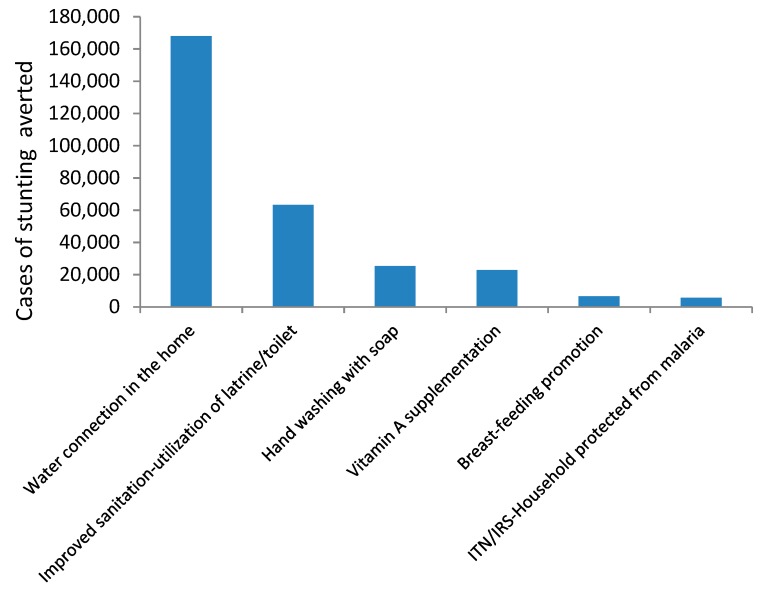
Number of stunting cases that would have been averted with scale-up of key interventions to coverage levels of the highest wealth quintile, 2019.

The scenario where intervention coverage is scaled up to the level of the highest wealth quintile highlights that closing inequalities in WASH-related interventions should be prioritized (Figure 6). For instance, scaling up water connection in homes to coverage levels of the highest wealth quintile can lead to about 168,000 cases of stunting being averted.

Using 2011 as a baseline year and projecting for 2016, the LiST estimated 42% of stunting relative to an observed 38% prevalence of stunting. About 30% of the observable changes in stunting prevalence were predicted by the tool.

## 4. Discussion

The present study highlights the remarkable progress that Ethiopia has made in stunting reduction between 2011 and 2016, but also indicates that current rates are unacceptably high. Inequalities by rural-urban and wealth quintile are observed, with the rural and the lower wealth quintiles being the most affected. While coverage of breastfeeding promotion and vitamin A supplementation were relatively high, interventions targeting (pregnant) women were limited in number and had particularly low coverage. A substantial increase in coverage of optimal complementary feeding, preventive zinc supplementation, and WASH-related interventions can avert a significant number of stunting cases. Addressing inequalities by wealth quintile, particularly for WASH interventions is also a key priority.

The higher stunting prevalence in rural settings and poorer households is not surprising and is consistent with literature on the social and environmental determinants of stunting [16,17,18]. Indeed, access to basic health services, markets, education, and basic infrastructure are much more limited in rural than in urban areas of Ethiopia [19,20]. More remote areas in Sub-Saharan Africa, including Ethiopia, also have worse linear growth and dietary outcomes [21]. Poorer households may also be more affected by high food prices and seasonality that can restrict their diets to predominantly starchy staples, with little or no consumption of nutrient-dense foods like animal-source foods, fruits and vegetables [22,23,24,25].

The low coverage of appropriate complementary feeding and the finding that the largest numbers of stunting cases are expected to be averted with universal scale-up of complementary feeding interventions is in line with the timing of stunting in Ethiopia [7]. In agreement with the worldwide timing of growth faltering, growth deficits significantly increased from 6–23 months, after which they stabilized [26]. Poor coverage of interventions that promote complementary feeding in remote areas, sub-optimal counseling skills of health-extension workers’ [27,28], as well as low availability, accessibility, and affordability of nutrient-dense foods can be related to the suboptimal complementary feeding practices [29]. This suggests that alignment of agricultural, food, and health systems is needed to significantly improve complementary feeding.

Coverage of interventions that address maternal nutrition was very low, which could partly explain the finding that the prevalence of stunting at birth in Ethiopia remained unchanged between 2000 and 2016 [7]. About 20% of children in rural areas and 10% in urban areas are stunted at birth, suggesting suboptimal maternal nutrition. Recent prospective cohort studies from Ethiopia have shown that maternal dietary diversity is low, partly due to food taboos and misperceptions [30]. Pregnant women who had lower dietary diversity had a significantly increased risk of adverse pregnancy outcomes including low birth weight [31,32]. This along with delayed first antenatal care visits and the low adherence to iron–folic acid supplementation can increase the risk of fetal growth restriction and thus calls for more attention to women’s and adolescent girl’s nutrition. With the growing evidence relating maternal diet to birth outcomes, monitoring the quality of women and adolescent’s diets by integrating indicators of dietary diversity into DHS and the LiST model could help inform the design of future food-based interventions.

On the other hand, addressing WASH-related inequalities and improving the coverage of water connection in homes, along with promotion of hand-washing, was identified as a priority action that, if addressed, can substantially reduce stunting cases. This finding is in contrast with recent findings from the SHINE and WASH Benefit trials that suggested no impact of WASH interventions on linear growth [33]. In the LiST stunting framework, WASH is not directly related to stunting, but is linked through diarrheal incidence pathways. Given that the relationship between frequent diarrheal incidence and stunting is well supported, increasing the reach and coverage of WASH services that are effective in reducing diarrheal incidence is critical [34]. However, to ensure that WASH activities reduce diarrhea incidence, effective interventions that extend beyond the traditional promotion of hand-washing with soap and access to clean water, to a “transformative WASH” that can also address fecal contamination in home environments is needed.

A number of limitations need to be considered when interpreting the findings of this study. First, effectiveness of the selected interventions in LiST comes from rigorous impact evaluations and systematic reviews of clinical trials that are delivered under controlled and ideal conditions and thus may provide slightly optimistic estimates. Second, the model does not capture the impact of interventions delivered outside the health sector, which is perhaps why only 30% of the observed change in stunting between 2011 and 2016 was predicted by the model. As data on the effectiveness of nutrition sensitive/supportive programs become available, integrating them to the LiST will be critical. While the estimates of coverage are drawn from updated and nationally representative data (i.e., DHS), data on coverage of some interventions were not available and thus were estimated using proxy indicators or had to be imputed from sub-national estimates. Finally, because of the national scope of this modeling, sub-national heterogeneity and disparities are not captured.

## 5. Conclusions

The present paper identified key interventions that if scaled-up can accelerate stunting reduction efforts in Ethiopia. A particular emphasis on increasing coverage of optimal complementary feeding, addressing zinc deficiencies through preventive zinc supplementation, and increasing the reach and coverage of WASH interventions is critical. Innovations in program delivery and health systems’ governance are required to effectively reach remote areas, rural communities, and the poorest. Such innovations need to be supported by well-designed nutrition-sensitive (supportive) programs and sustained and equitable economic growth.

## Supplementary Materials

The following are available online at https://www.mdpi.com/2072-6643/11/12/3065/s1, File S1: Data sources.
