# Supplementary material for: Prioritizing the Scale-Up of Evidence-Based Nutrition and Health Interventions to Accelerate Stunting Reduction in Ethiopia
Source: Nutrients. 2019 Dec 16;11(12):3065. doi: 10.3390/nu11123065 (PMC6950157; doi:10.3390/nu11123065)
Supplement: Supplementary file 1 [file nutrients-11-03065-s001.pdf]

Supplementary file S1: Data sources

1. Total population

Summary: Total population: Estimated to be consistent with the 1984, 1994 and 2007 censuses adjusted for underenumeration, and with estimates of the subsequent trends in fertility, mortality and international migration.

Source: Census Date: 28-May-2007

2. Total fertility rate

Summary: Total fertility: Based on: (a) maternity-history data from the 2000, 2005, 2011 and preliminary results from the 2016 Ethiopia Demographic and Health Survey (DHS), and the 1990 National Family and Fertility Survey (NFFS); (b) data on births during the past 12 months, classified by age of mother, from the 1984, 1994 and 2007 censuses, the 1964 National Sample Survey (NSS), the 1st and 2nd rounds of the 1969/71 NSS, the 1981 Demographic Survey; (c) data on last births, classified by age of mother, from the round 1 and 2 of the 2014 Performance and Monitoring Accountability 2020 (PMA2020); and (d) indirect estimates obtained from the application of the reverse survival method to the 1981 Demographic Survey, the 2003 World Health Survey (WHS) and the 1994 and 2007 censuses.

3. Coverage data

**Intervention**	**Data source**
Iron supplementation in pregnancy	DHS 2011, DHS 2016
Multiple micronutrient supplementation	Not implemented
Balanced energy-supplementation	Not implemented
Calcium supplementation in pregnancy	Not implemented
Complementary feeding - education only	DHS 2011, DHS 2016
Complementary feeding - supplementary feeding and education	DHS 2011, DHS 2016
Vitamin A supplementation	UNICEF Vitamin A supplementation data (updated April 2018, currently available for 2000-2016)
Zinc supplementation	Not implemented
Improved water source	Source for all years: WHO/UNICEF Joint Monitoring Program on Water and Sanitation (JMP) - https://washdata.org/data; updated 2017 (data available through 2015)
Water connection in the home	Source for all years: WHO/UNICEF Joint Monitoring Program on Water and Sanitation (JMP) - https://washdata.org/data; updated 2017 (data available through 2015)
Improved sanitation - Utilization of latrines or toilets	Source for all years: WHO/UNICEF Joint Monitoring Program on Water and Sanitation (JMP) - https://washdata.org/data; updated 2017 (data available through 2015)
Hand washing with soap	DHS 2011, DHS 2016
Hygienic disposal of children's stools	DHS 2011, DHS 2016
ITN/IRS - Households protected from malaria	DHS 2005 (ITN only), MIS 2015* (ITN only)

Rotavirus vaccine	WHO-UNICEF coverage estimates series, http://apps.who.int/immunization_monitoring/globalsummary/timeseries/tswucoveragerotac.html; dataset last updated 15 July 2018
KMC - Kangaroo mother care	DHS 2016 (proxies are used)
Zinc for treatment of diarrhea	DHS 2011, DHS 2016
Breastfeeding prevalence	DHS 2011, DHS 2016
Prevalence of early initiation of breastfeeding	DHS 2011, DHS 2016

4. Source of effectiveness/impact estimates

Impacts on stunting

Term AGA

Odds ratio source: LiST Technical Note. https://static1.squarespace.com/static/5bbba6574d8711a7dcafa92a/t/5c93e9b9f4e1fc34bdbe9cc8/1553197497868/Birth+outcomes+on+stunting+at+1+mo.pdf

Term SGA

Odds ratio source: LiST Technical Note. https://static1.squarespace.com/static/5bbba6574d8711a7dcafa92a/t/5c93e9b9f4e1fc34bdbe9cc8/1553197497868/Birth+outcomes+on+stunting+at+1+mo.pdf

Pre-term AGA

Odds ratio source: LiST Technical Note. https://static1.squarespace.com/static/5bbba6574d8711a7dcafa92a/t/5c93e9b9f4e1fc34bdbe9cc8/1553197497868/Birth+outcomes+on+stunting+at+1+mo.pdf

Pre-term SGA

Odds ratio source: LiST Technical Note. https://static1.squarespace.com/static/5bbba6574d8711a7dcafa92a/t/5c93e9b9f4e1fc34bdbe9cc8/1553197497868/Birth+outcomes+on+stunting+at+1+mo.pdf

Not stunted at previous age cohort

Odds ratio source: Cousens S, Perin J, Christian P, et al. Modelling stunting in LiST: the effect of applying smoothing to linear growth data. BMC Public Health 2017, 17(4S):778. https://doi.org/10.1186/s12889-017-4744-3

Stunted at previous age cohort

Odds ratio source: Cousens S, Perin J, Christian P, et al. Modelling stunting in LiST: the effect of applying smoothing to linear growth data. BMC Public Health 2017, 17(4S):778. https://doi.org/10.1186/s12889-017-4744-3

Food secure with promotion

Odds ratio source: Panjwani A and Heidkamp R. Complementary Feeding Interventions Have a Small but Significant Impact on Linear and Ponderal Growth of Children in Low- and Middle-Income Countries: A Systematic Review and Meta-Analysis. J Nutr 2017, 147(11S):2169S-2178S. https://doi.org/10.3945/jn.116.243857

Food secure without promotion

Odds ratio source: Panjwani A and Heidkamp R. Complementary Feeding Interventions Have a Small but Significant Impact on Linear and Ponderal Growth of Children in Low- and Middle-Income Countries: A Systematic Review and Meta-Analysis. J Nutr 2017, 147(11S):2169S-2178S. https://doi.org/10.3945/jn.116.243857

Insecure with promotion and supplementation

Odds ratio source: Panjwani A and Heidkamp R. Complementary Feeding Interventions Have a Small but Significant Impact on Linear and Ponderal Growth of Children in Low- and Middle-Income Countries: A Systematic Review and Meta-Analysis. J Nutr 2017, 147(11S):2169S-2178S. https://doi.org/10.3945/jn.116.243857

Insecure with neither promotion nor supplementation

Odds ratio source: Panjwani A and Heidkamp R. Complementary Feeding Interventions Have a Small but Significant Impact on Linear and Ponderal Growth of Children in Low- and Middle-Income Countries: A Systematic Review and Meta-Analysis. J Nutr 2017, 147(11S):2169S-2178S. https://doi.org/10.3945/jn.116.243857

Impact of diarrhea per episode

Odds ratio source: Checkley W, Buckley G, Gilman RH, et al. Multi-country analysis of the effects of diarrhoea on childhood stunting. Int J Epidemiol 2008. https://www.ncbi.nlm.nih.gov/pubmed/18567626

Zinc supplemented

Odds ratio source: Bhutta ZA, Das JK, Rizvi A, et al. Evidence-based interventions for improvement of maternal and child nutrition: what can be done and at what cost? Lancet 2013; 382(9890): 352-77. http://www.ncbi.nlm.nih.gov/pubmed/23746776 (Supplemental material, page 16.)

Not zinc supplemented

Odds ratio source: Bhutta ZA, Das JK, Rizvi A, et al. Evidence-based interventions for improvement of maternal and child nutrition: what can be done and at what cost? Lancet 2013; 382(9890): 352-77. http://www.ncbi.nlm.nih.gov/pubmed/23746776 (Supplemental material, page 16.)

Impacts on maternal anemia

Iron supplementation in pregnancy

Effectiveness source: Pena-Rosas JP, De-Regil LM, Garcia-Casal MN, et al. Daily oral iron supplementation during pregnancy. Cochrane Database Syst Rev 2015. http://www.ncbi.nlm.nih.gov/pubmed/26198451

Multiple micronutrient supplementation in pregnancy

Effectiveness source: Pena-Rosas JP, De-Regil LM, Garcia-Casal MN, et al. Daily oral iron supplementation during pregnancy. Cochrane Database Syst Rev 2015. http://www.ncbi.nlm.nih.gov/pubmed/26198451

Blanket iron supplementation/fortification

Effectiveness source: Low MS, Speedy J, Styles CE, et al. Daily iron supplementation for improving anaemia, iron status and health in menstruating women. Cochrane Database Syst Rev 2016. http://www.ncbi.nlm.nih.gov/pubmed/27087396

Pregnant women protected via IPTP

Effectiveness source: Radeva-Petrova D, Kayentao K, Ter Kuile FO, et al. Drugs for preventing malaria in pregnant women in endemic areas: Any drug regimen versus placebo or no treatment. Cochrane Database Syst Rev 2014. http://www.ncbi.nlm.nih.gov/pmc/articles/PMC4498495/

ITN/IRS - Households protected from malaria

Effectiveness source: Radeva-Petrova D, Kayentao K, Ter Kuile FO, et al. Drugs for preventing malaria in pregnant women in endemic areas: Any drug regimen versus placebo or no treatment. Cochrane Database Syst Rev 2014. http://www.ncbi.nlm.nih.gov/pmc/articles/PMC4498495/

Impact of promotion on age-appropriate breastfeeding

Sinha B, Chowdhury R, Prakash Upadhyay R, Taneja S, Martines J, Bahl R, Jeeva Sankar M; Integrated Interventions Delivered in Health Systems, Home, and Community Have the Highest Impact on Breastfeeding Outcomes in Low- and Middle-Income Countries. Journal Nutr 2017. 147(11S): 2179S–2187S, https://doi.org/10.3945/jn.116.242321

Sinha B, Chowdhury R, Prakash Upadhyay R, Taneja S, Martines J, Bahl R, Jeeva Sankar M; Integrated Interventions Delivered in Health Systems, Home, and Community Have the Highest Impact on Breastfeeding Outcomes in Low- and Middle-Income Countries. Journal Nutr 2017. 147(11S): 2179S–2187S, https://doi.org/10.3945/jn.116.242321

Percent of children who are LBW

Percent of children who are LBW

Kozuki N, Lee AC, Silveira MF, Victora CG, Adair L, Humphrey J, et al. The associations of birth intervals with small-for-gestational-age, preterm, and neonatal and infant mortality: a meta-analysis. BMC Public Health. 2013;13 Suppl 3:S3.

Kangaroo mother care on breastfeeding

Odds ratio source: Boundy EO, Dastjerdi R, Spiegelman D, et al. Kangaroo mother care and neonatal outcomes: A meta-analysis. Pediatrics 2016; 137(1). http://www.ncbi.nlm.nih.gov/pubmed/26702029

Impacts on diarrhea incidence

Improved sanitation - Utilization of latrines or toilets

Effectiveness source: Meta-analysis of 3 studies by Christa Fischer-Walker. (publication forthcoming). (1) Aziz KMA, Hoque BA, Hasan KZ, Patwary MY, Huttly SRA, Rahman MM, et al. Reduction in diarrhoeal diseases in children in rural Bangladesh by environmental and behavioural modifications. Transactions of the Royal Society of Tropical Medicine and Hygiene 1990;84(3):433-8. (2) Garrett V, Ogutu P, Mabonga P, et al. Diarrhoea prevention in a high-risk rural Kenyan population through point-of-use chlorination, safe water storage, sanitation, and rainwater harvesting. Epidemiology and Infection 2008;136(11):1463-71. (3) Messou E, Sangare SV, Josseran R, Le Corre C, Guelain J. Effect of hygiene and water sanitation and oral rehydration on diarrhoea and mortality of children children less than five years old in the south of Ivory Coast. Bulletin de la Société de Pathologie Exotique 1997;90(1):44-7.

Improved water source

Effectiveness source: Meta-analysis of 3 studies by Christa Fischer-Walker. (publication forthcoming). (1) Aziz KMA, Hoque BA, Hasan KZ, Patwary MY, Huttly SRA, Rahman MM, et al. Reduction in diarrhoeal diseases in children in rural Bangladesh by environmental and behavioural modifications. Transactions of the Royal Society of Tropical Medicine and Hygiene 1990;84(3):433-8. (2) Garrett V, Ogutu P, Mabonga P, et al. Diarrhoea prevention in a high-risk rural Kenyan population through point-of-use chlorination, safe water storage, sanitation, and rainwater harvesting. Epidemiology and Infection 2008;136(11):1463-71. (3) Messou E, Sangare SV, Josseran R, Le Corre C, Guelain J. Effect of hygiene and water sanitation and oral rehydration on diarrhoea and mortality of children children less than five years old in the south of Ivory Coast. Bulletin de la Société de Pathologie Exotique 1997;90(1):44-7.

Water connection in the home

Effectiveness source: Cairncross S, Valdmanis V. Water supply, sanitation, and hygiene promotion. In: Jamison DT, Breman JG, Measham AR, et al., editors. Disease control priorities in developing countries. Washington DC: The World Bank, 2006; p. 771-792. http://www.ncbi.nlm.nih.gov/books/NBK11728/

Hand washing with soap

Effectiveness source: Darvesh et al. Water, sanitation and hygiene interventions for acute childhood diarrhea: a systematic review to provide estimates for the Lives Saved Tool. BMC Public Health. 2017 Nov 7;17(Suppl 4):776. doi: 10.1186/s12889-017-4746-1.

Hygienic disposal of children's stools

Effectiveness source: Clasen et al. Interventions to improve disposal of human excreta for preventing diarrhoea. Cochrane. 2010.

Zinc supplementation

Effectivness source: Yakoob MY, Theodoratou E, Jabeen A, et al. Preventive zinc supplementation in developing countries: impact on mortality and morbidity due to diarrhea, pneumonia and malaria. BMC Public Health 2011; 11(Suppl 3): S23. http://www.ncbi.nlm.nih.gov/pubmed/21501441.

Black RE, Victora CG, Walker SP, et al. Maternal and child undernutrition and overweight in low-income and middle-income countries. Lancet 2013; 382(9890): 427-51. (Supplemental material, page 18-19.) http://www.ncbi.nlm.nih.gov/pubmed/23746772

Vitamin A supplementation

Effectiveness source: Imdad A, Yakoob MY, Sudfeld CR, et al. Impact of vitamin A supplementation on infant and childhood mortality. BMC Public Health 2011; 11(Suppl 3): S20. http://www.ncbi.nlm.nih.gov/pubmed/21501438

Age and birth order: impacts on birth outcomes

All ages

Relative risk source: Kozuki N, Lee ACC, Silveira MF, et al. The associations of parity and maternal age with small-for-gestational-age, preterm, and neonatal and infant mortality: a meta-analysis. BMC Public Health 2013; 13(Suppl 3): S2. http://www.ncbi.nlm.nih.gov/pmc/articles/PMC3847520/. (Relative risks are from unpublished calculations associated with the paper.)

Less than 18 years

Relative risk source: Kozuki N, Lee ACC, Silveira MF, et al. The associations of parity and maternal age with small-for-gestational-age, preterm, and neonatal and infant mortality: a meta-analysis. BMC Public Health 2013; 13(Suppl 3): S2. http://www.ncbi.nlm.nih.gov/pmc/articles/PMC3847520/. (Relative risks are from unpublished calculations associated with the paper.)

18 - 34 years old

Relative risk source: Kozuki N, Lee ACC, Silveira MF, et al. The associations of parity and maternal age with small-for-gestational-age, preterm, and neonatal and infant mortality: a meta-analysis. BMC Public Health 2013; 13(Suppl 3): S2. http://www.ncbi.nlm.nih.gov/pmc/articles/PMC3847520/. (Relative risks are from unpublished calculations associated with the paper.)

All ages

Relative risk source: Kozuki N, Lee ACC, Silveira MF, et al. The associations of parity and maternal age with small-for-gestational-age, preterm, and neonatal and infant mortality: a meta-analysis. BMC Public

Health 2013; 13(Suppl 3): S2. http://www.ncbi.nlm.nih.gov/pmc/articles/PMC3847520/. (Relative risks are from unpublished calculations associated with the paper.)

Less than 18 years

Relative risk source: Kozuki N, Lee ACC, Silveira MF, et al. The associations of parity and maternal age with small-for-gestational-age, preterm, and neonatal and infant mortality: a meta-analysis. BMC Public Health 2013; 13(Suppl 3): S2. http://www.ncbi.nlm.nih.gov/pmc/articles/PMC3847520/. (Relative risks are from unpublished calculations associated with the paper.)

18 - 34 years old

Relative risk source: Kozuki N, Lee ACC, Silveira MF, et al. The associations of parity and maternal age with small-for-gestational-age, preterm, and neonatal and infant mortality: a meta-analysis. BMC Public Health 2013; 13(Suppl 3): S2. http://www.ncbi.nlm.nih.gov/pmc/articles/PMC3847520/. (Relative risks are from unpublished calculations associated with the paper.)

All ages

Relative risk source: Kozuki N, Lee ACC, Silveira MF, et al. The associations of parity and maternal age with small-for-gestational-age, preterm, and neonatal and infant mortality: a meta-analysis. BMC Public Health 2013; 13(Suppl 3): S2. http://www.ncbi.nlm.nih.gov/pmc/articles/PMC3847520/. (Relative risks are from unpublished calculations associated with the paper.)

Less than 18 years

Relative risk source: Kozuki N, Lee ACC, Silveira MF, et al. The associations of parity and maternal age with small-for-gestational-age, preterm, and neonatal and infant mortality: a meta-analysis. BMC Public Health 2013; 13(Suppl 3): S2. http://www.ncbi.nlm.nih.gov/pmc/articles/PMC3847520/. (Relative risks are from unpublished calculations associated with the paper.)

18 - 34 years old

Relative risk source: Kozuki N, Lee ACC, Silveira MF, et al. The associations of parity and maternal age with small-for-gestational-age, preterm, and neonatal and infant mortality: a meta-analysis. BMC Public Health 2013; 13(Suppl 3): S2. http://www.ncbi.nlm.nih.gov/pmc/articles/PMC3847520/. (Relative risks are from unpublished calculations associated with the paper.)

Birth intervals: impacts on birth outcomes

First birth

Relative risk source: Kozuki N, Lee ACC, Silveira MF, et al. The associations of birth intervals with small-for-gestational-age, preterm, and neonatal and infant mortality: a meta-analysis. BMC Public Health 2013; 13(Suppl 3): S3. http://www.ncbi.nlm.nih.gov/pmc/articles/PMC3847557/. (Relative risks are from unpublished calculations associated with the paper.)

less than 18 months

Relative risk source: Kozuki N, Lee ACC, Silveira MF, et al. The associations of birth intervals with small-for-gestational-age, preterm, and neonatal and infant mortality: a meta-analysis. BMC Public Health 2013; 13(Suppl 3): S3. http://www.ncbi.nlm.nih.gov/pmc/articles/PMC3847557/. (Relative risks are from unpublished calculations associated with the paper.)

18-23 months

Relative risk source: Kozuki N, Lee ACC, Silveira MF, et al. The associations of birth intervals with small-for-gestational-age, preterm, and neonatal and infant mortality: a meta-analysis. BMC Public Health 2013; 13(Suppl 3): S3. http://www.ncbi.nlm.nih.gov/pmc/articles/PMC3847557/. (Relative risks are from unpublished calculations associated with the paper.)

24 months or greater

Relative risk source: Kozuki N, Lee ACC, Silveira MF, et al. The associations of birth intervals with small-for-gestational-age, preterm, and neonatal and infant mortality: a meta-analysis. BMC Public Health 2013; 13(Suppl 3): S3. http://www.ncbi.nlm.nih.gov/pmc/articles/PMC3847557/. (Relative risks are from unpublished calculations associated with the paper.)

Maternal nutrition

IPTp - Intermittent preventive treatment of malaria during pregnancy

Effectiveness source: Eisele TP, Larsen D, Steketee RW. Protective efficacy of interventions for preventing malaria mortality in children in Plasmodium falciparum endemic areas. International Journal of Epidemiology 2010; 39(Suppl 1): i88-i10. http://www.ncbi.nlm.nih.gov/pmc/articles/PMC2845865/ (The effect size for ITN usage is used as a proxy for IPTp.)

Balanced energy supplementation

Effectiveness source: Imdad A, Butta Z. Effect of balanced protein energy supplementation during pregnancy on birth outcomes. BMC Public Health 2011, (Suppl 3): S17. DOI: 10.1186/1471-2458-11-S3-S17

Ota E, Hori H, Mori R, et al. Antenatal dietary education and supplementation to increase energy and protein intake. Cochrane Database Syst Rev 2015. http://www.ncbi.nlm.nih.gov/pubmed/26031211.

Iron supplementation in pregnancy

Effectiveness source: Haider BA, Olofin I, Wang M, et al. Anaemia, prenatal iron use, and risk of adverse pregnancy outcomes: systematic review and meta-analysis. BMJ. 2013;346:f3443. https://www.ncbi.nlm.nih.gov/pubmedhealth/PMH0057254/. See also: LiST Technical Note. http://livessavedtool.org/images/documents/Technical_Notes/Iron-and-MMN-effect-sizes.pdf

Multiple micronutrient supplementation in pregnancy (low BMI women)

Effectiveness source: Smith ER, Shankar AH, Wu LSF, et al. Modifiers of the effect of maternal multiple micronutrient supplementation on stillbirth, birth outcomes, and infant mortality: a meta-analysis of individual patient data from 17 randomised trials in low-income and middle-income countries. Lancet Global Health 2017; 5(11): e1090-e1100. http://www.thelancet.com/journals/langlo/article/PIIS2214-109X(17)30371-6/fulltext

Multiple micronutrient supplementation in pregnancy (healthy BMI)

Effectiveness source: Smith ER, Shankar AH, Wu LSF, et al. Modifiers of the effect of maternal multiple micronutrient supplementation on stillbirth, birth outcomes, and infant mortality: a meta-analysis of individual patient data from 17 randomised trials in low-income and middle-income countries. Lancet Global Health 2017; 5(11): e1090-e1100. http://www.thelancet.com/journals/langlo/article/PIIS2214-109X(17)30371-6/fulltext

Calcium supplementation

Effectiveness source: Imdad A, Jabeen A, Bhutta, ZA. Role of calcium supplementation during pregnancy in reducing risk of developing gestational hypertensive disorders: a meta-analysis of studies from developing countries. BMC Public Health 2011; 11(Suppl 3): S18. http://www.ncbi.nlm.nih.gov/pmc/articles/PMC3231891/

ITN/IRS - Households protected from malaria

Effectiveness source: Eisele TP, Larsen D, Steketee RW. Protective efficacy of interventions for preventing malaria mortality in children in Plasmodium falciparum endemic areas. International Journal of Epidemiology 2010; 39(Suppl 1): i88-i10. http://www.ncbi.nlm.nih.gov/pmc/articles/PMC2845865/
